# Evaluation of the Ability of PAMPA Membranes to Emulate Biological Processes through the Abraham Solvation Parameter Model

**DOI:** 10.3390/membranes13070640

**Published:** 2023-06-30

**Authors:** Sara Soriano-Meseguer, Elisabet Fuguet, Adriana Port, Martí Rosés

**Affiliations:** 1 Departament d’Enginyeria Química i Química Analítica, Universitat de Barcelona (UB), 08028 Barcelona, Spain; sara.sorioanomeseguer@gmail.com (S.S.-M.); marti.roses@ub.edu (M.R.); 2 Institut de Biomedicina de la Universitat de Barcelona (IBUB), Universitat de Barcelona (UB), 08028 Barcelona, Spain; 3 Serra Húnter Programme, Generalitat de Catalunya, 08002 Barcelona, Spain; 4 Welab Barcelona, Parc Científic de Barcelona, 08028 Barcelona, Spain; aport@welab.barcelona

**Keywords:** skin permeation, human intestinal absorption, blood-brain permeation, blood-brain partition, PAMPA, solvation parameter model, prediction

## Abstract

Two parallel artificial membrane permeability assay (PAMPA) systems intended for emulating skin permeability have been characterized through the solvation parameter model of Abraham using multilinear regression analysis. The coefficients of the obtained equations have been compared to the ones already established for other PAMPA membranes using statistical tools. The results indicate that both skin membranes are similar to each other in their physicochemical properties. However, they are different from other PAMPA membranes (e.g., intestinal absorption and blood–brain PAMPAs), mainly in terms of hydrophobicity and hydrogen bonding properties. Next, all PAMPA membranes have been compared to relevant biological processes also characterized through the solvation parameter model. The results highlight that skin-PAMPA membranes are a very good choice to emulate skin permeability.

## 1. Introduction

During the last two decades, several systems based on parallel artificial membrane permeability assays (PAMPA) have been developed for the estimation of biological permeabilities. Since the first system developed by Kansy et al. [1], many PAMPA-based systems have emerged with the aim of emulating the permeation of compounds via passive diffusion through biological membranes. Initially, most of the membranes were developed to simulate the intestinal absorption (IA) of compounds [2,3,4]. Different lipidic mixtures have been used for this purpose. Some of these consist of simple systems like a phospholipid dissolved in a non-polar solvent, such as n-dodecane [5], whereas other systems use more complex mixtures [6,7]. In fact, the composition of the lipidic mixture in the PAMPA membrane has a direct impact of the obtained permeability values and this has been a key factor for the targeted development of systems that emulate specific biological membranes [3,8]. This is the case, for example, of systems developed to mimic brain [9,10,11] or skin permeation [12,13].

The usual way to test whether a given PAMPA system mimics adequately a certain biological permeability process is to correlate the available in vitro permeability data with the corresponding permeabilities obtained in the PAMPA system. However, the direct correlation between the two properties only provides information on the similarities between the two systems involved in the correlation. Another way to compare how similar a large number of systems are is by means of the characterization of all of them through a common model and the further evaluation of the obtained equations. In this way, with adequate comparison tools, a high number of systems of very different nature (PAMPA, biological, chromatographic, etc.) can be compared all together at once by means of the corresponding coefficients of the common model. Thus, a wider vision between the similarities and differences between all the compared systems is obtained. The solvation parameter model, developed by M. H. Abraham, has been widely used for this purpose [14]. This model is based on linear free energy relationships (LFER) and correlates a dependent variable (for example a partition coefficient, a chromatographic retention factor, or the permeability of a given neutral compound among many other physicochemical and biological systems) with a series of descriptors of the compound, by means of Equation (1):

(1) logSP=c+eE+sS+aA+bB+vV

In this equation, *SP* is a solute property related to a free energy change. In the case of PAMPA systems, the property would be the intrinsic permeability (*P*_0_). The independent variables are *E*, *S*, *A*, *B*, and *V*. These are descriptors of the solute related to: *E*, the excess molar refractivity of the solute compared to the one of an alkane with the same number of C atoms (in cm^3^ mol^−1^/10); *S*, its dipolarity/polarizability; *A*, the solute’s hydrogen bond acidity; *B*, its hydrogen bond basicity; and *V*, its McGowan volume (in cm^3^ mol^−1^/100). The coefficients of the equation are obtained using the multiple linear correlation of the log *SP* property for a set of compounds to their corresponding descriptor values, and the magnitude and sign of the coefficients provide information on the chemistry of the evaluated system. This equation has been indistinctly applied to numerous solvent partition systems [15,16], to chromatographic partition systems [17,18,19,20], and also to many environmental [21,22,23,24,25] and biological systems [26,27,28,29,30,31]. This implies an important volume of systems that can be compared, in order to identify similarities between them through the coefficients of the equation. For example, complex systems like tadpole narcosis [32], aquatic toxicity [33,34], or even skin permeation [35] have been satisfactorily emulated through simple chromatographic systems after a careful analysis and comparison of the coefficients for the respective equations [36].

In this sense, in 2015, Abraham [37] used his model to establish the equations for a series of PAMPA permeation systems and compared the coefficients of PAMPA equations with those of other systems. He chose solvent–solvent partition systems as well as biological systems related to the distribution of compounds across a membrane, like the human intestinal absorption (HIA), the permeation of the brain barrier, and the permeation of the skin. He concluded that, of the studied systems, only the PAMPA-P16 with a hexadecane membrane [38] would be a good model for HIA, and none of the systems appeared to be good enough to model brain or skin permeation. Although specific PAMPA membranes for skin had been developed by that time [12,13], there was not enough permeability data available for its evaluation through Equation (1). Today, there are more data available for skin-PAMPA systems. On one hand, in a previous work in which the dependence of the effective permeability (*P*_e_) with pH was evaluated [39], the permeability of a series of nearly 50 neutral compounds in a commercial skin-PAMPA (PAMPA-Certramide) membrane composed by certramide, cholesterol, stearic acid, and silicon oil was measured [13]. On the other hand, Ottaviani et al. also measured the permeability of a series of compounds in a membrane composed of 70% silicone and 30% isopropyl myristate (IPM) [12] with the aim of emulating skin permeation (PAMPA-IPM). Thus, the objective of the present work is to model the existing skin-PAMPA systems through Equation (1) and evaluate the similarities both with other PAMPA membranes and with relevant biological systems related to distribution across a membrane.

## 2. Materials and Methods

### 2.1. Instruments

pH measurements were carried out in a Crison 2001 pH meter from Hach Lange Spain, (L’Hospitalet de Llobregat, Spain), using a Crison 5202 combined electrode. The electrode system was calibrated with ordinary aqueous buffers at pH 4.01 and 7.00 (25 °C).

A PAMPA Explorer Permeability Assay instrument from Pion Inc. (Billerica, MA, USA), with a Gut-BoxTM and a TempPlate, was used for the skin-PAMPA permeability measurements. The Gut-BoxTM is used to reduce the unstirred water layer thickness, and the TempPlate is used for temperature control during plate incubation.

To quantify the amount of test compounds in the donor and acceptor compartments, a Waters (Milford, MA, USA) I-Class UPLC with diode array detector and an Acquity UPLC BEH C18 (50 × 2.1 mm, 1.7 μm) column also from Waters were used. Instrument control and data processing was performed through the software Empower 3.

### 2.2. Reagents

Acetonitrile LiChrosolv grade and 0.5 M sodium hydroxide solution were purchased from Merck (Darmstadt, Germany). Formic acid was obtained from Scharlau (Sentmenat, Spain). Dimethylsulphoxide was from Carlo Erba (Milano, Italy). Water was purified by a Milli-Q deionizing system from Millipore (Billerica, MA, USA) with a resistivity of 18.2 MΩ. Most solutes employed were purchased from Sigma-Aldrich (Steinheim, Germany), Fluka Analytical VWR (West Chester, PA, USA), Riedel-de Haën (Seelze, Germany), Merck (Darmstadt, Germany), Carlo Erba (Milano, Italy), and Baker (Center Valley, PA, USA).

A concentrated PRISMA HTTM solution from Pion Inc. (Billerica, MA, USA) was used to prepare the buffer solutions for PAMPA experiments. It consists of a universal buffer composed of several compounds with evenly spaced p*K*_a_ values to produce a constant buffer capacity in the range pH 3–10. Its ionic strength is about 10 mM. The skin-PAMPA plates were also obtained from Pion Inc. and a hydration solution from the same supplier was used to rehydrate the artificial skin membrane.

### 2.3. Skin-PAMPA P_e_ Determination

First, the top part of the skin-PAMPA (PAMPA-Certramide) sandwich, which contains the membrane, was hydrated overnight with the hydration solution. Different buffer solutions at pH 3, 4, 5, 6, 7, and 7.4 were prepared to solve the samples. To prepare them, 25 mL of concentrated PRISMA HTTM was diluted to 1 L with water, and 0.5 M NaOH was added to obtain the desired pH. Above pH 8, the skin-PAMPA membrane becomes degraded [40], so pH 7.4 was the highest pH used in the present work. In order to measure the intrinsic permeability, each sample was dissolved in the buffer solution with a pH that ensured the presence of the neutral form. The concentration of the drug sample solutions was 50 µM, and all samples contained 0.5% *v*/*v* dimethylsulfoxide. In all instances, the pH of the acceptor compartment was 7.4. The list of analyzed compounds is available in Table S1 of the Supplementary Materials.

A total of 180 µL of sample solution was placed in the donor compartment, and 200 µL of pH 7.4 buffer was placed in the acceptor compartment. The lower volume in the donor compartment was due to the presence of stirring bars, which already had a volume of 20 µL.

According to previous studies, in which the conditions in the skin-PAMPA measurements were optimized [40], the skin-PAMPA sandwich was incubated at 32 °C for 4 h. Afterwards, the concentration of the compounds was measured in both the donor and the acceptor plates. Additionally, the initial sample solution was quantified. This quantification was carried out by UPLC-DAD, using 0.1% formic acid and acetonitrile as mobile phase, a flow rate of 0.8 mL/min, and a linear gradient elution from 2% to 98% of acetonitrile in 2.5 min. The injection volume was 5 μL, and 3 to 5 replicate measurements were performed per compound. Every well-plate contained only one compound.

### 2.4. Permeability Data Treatment

The skin-PAMPA permeability was calculated through the following equations [4]:

(2) $$P_{e}=-\frac{2{.303V}_{D}}{A\cdot \left({t-t}_{\mathrm{ss}}\right){\cdot \varepsilon }_{a}}\cdot \left(\frac{1}{{1+r}_{a}}\right){\cdot \log}_{10}\left[-{\;r}_{a}+\left(\frac{{1+r}_{a}}{{1-R}_{M}}\right)\cdot \frac{C_{D}\left(t\right)}{C_{D}\left(0\right)}\right]$$

(3) $$R_{M}=1-\frac{C_{D}\left(t\right)}{C_{D}\left(0\right)}-\frac{V_{A}}{V_{D}}\frac{C_{A}\left(t\right)}{C_{D}\left(0\right)}$$

(4) $$t_{\mathrm{ss}}=\left(54R_{M}+1\right)\cdot 60$$

where *V*_D_ and *V*_A_ are the volumes of solution on the donor (0.18 cm^3^) and acceptor (0.2 cm^3^) sides, respectively, *A* is the area of the membrane (0.3 cm^2^), *t* is the incubation time of the experiment (s), *t*_ss_ is the lag time (s), *ε*_a_ is the apparent membrane porosity (0.76), *C*_D_(*t*) is the concentration (mol cm^−3^) on the donor side at time *t*, *C*_D_(0) is the initial concentration (mol cm^−3^) on the donor side, *C*_A_(*t*) is the concentration (mol cm^−3^) on the acceptor side at time *t*, *RM* is the membrane retention factor, and *r*_a_ is the asymmetry ratio, defined as:

(5) $$r_{a}=\left(\frac{V_{D}}{V_{A}}\right)\frac{P_{e(A\to D)}}{P_{e(D\to A)}}$$

When the pH is different on the two sides of the membrane, a gradient-pH is created and the permeation of ionizable molecules can be altered. This gradient-pH implies two different permeability coefficients, one on each direction of the membrane, denoted by the subscript (D→A) or (A→D). Equation (5) has two unknowns, *P*_e(A→D)_ and *P*_e(D→A)_, so an iterative method has to be used to solve it. To this purpose, two different experiments were carried out for each compound, one with a gradient-pH and the other with an iso-pH, that is, the same pH in both compartments (7.4). For the iso-pH, *P*_e(A→D)_ = *P*_e(D→A)_. Therefore, *P*_e(A→D)_ can be solved directly using the iso-pH equation:

(6) $$P_{e}=-\frac{2{.303V}_{D}}{A\cdot \left({t-t}_{\mathrm{ss}}\right){\cdot \varepsilon }_{a}}\cdot \left(\frac{1}{{1+r}_{v}}\right){\cdot \log}_{10}\left[-r_{v}+\left(\frac{{1+r}_{v}}{1-R_{M}}\right)\cdot \frac{C_{D}\left(t\right)}{C_{D}\left(0\right)}\right]$$

where *r*_v_ is the volume ratio in the aqueous compartment:

(7) $$r_{v}=\frac{V_{D}}{V_{A}}$$

Finally, Equation (5) was iteratively solved for *P*_e(D→A)_. Initially, *r*_a_ was assumed to be *r*_v_, but the *r*_a_ value was improved after each iteration by using the newly obtained *P*_e(D→A)_. The process continued until self-consistency was reached within the precision required (0.001). The Solver tool in Microsoft Excel was used for the iterative process.

### 2.5. Calculation of the Similarity between Systems

The similarity between systems characterized by a common LFER model can be evaluated via the Euclidean distance between the LFER coefficients of the systems. There are several ways to calculate this parameter, although in all cases, the smaller the distance value, the more mathematically similar the two compared systems. Abraham and Martins [28] introduced the *D*′, which is based on five-dimensional vectors that take each coefficient of the equation as one dimension. It evaluates the difference between systems via the subtraction of the coefficients. In the case of Equation (1), *D*′ is calculated as follows:

(8) $$D’=\sqrt{{\left(e_{i}-e_{j}\right)}^{2}+{\left(s_{i}-s_{j}\right)}^{2}+{\left(a_{i}-a_{j}\right)}^{2}+{\left(b_{i}-b_{j}\right)}^{2}+{\left(v_{i}-v_{j}\right)}^{2}}$$

where the subscripts *i* and *j* refer to the systems that are compared.

### 2.6. Data Analysis

All correlations were performed using Microsoft Excel, and the experimental descriptor values for the compounds were obtained via Percepta software (ACDLabs, Toronto, ON, Canada) [41]. PCA and dendrogram analysis were carried out using Past 4.03 [42].

## 3. Results and Discussion

### 3.1. Characterization of Skin-PAMPA Systems through the Solvation Parameter Model

Ottaviani et al. [12] designed different artificial membranes based on silicone oil and isopropyl myristate to model passive human skin permeation. Among the studied compositions, the one that had better correlations between skin permeation (*K*_p_) and *P*_e_ values was the one with 70% silicon oil and 30% IPM. They tested the permeability of 31 compounds in iso-pH conditions and selected, for each compound, a pH in which the ionized fraction was lower than 0.2. However, as Equation (1) accounts for neutral compounds, compounds with a degree of ionization higher than 0.02 were excluded. Table S2 of the supplementary material shows the final set of 27 compounds used for the correlation, the log *P*_e_ values, and the experimental descriptor values. Equation (9) shows the obtained results of the multilinear regression:

log *P*_e_ (PAMPA-IPM) = −4.202 (0.195) + 0.081 (0.216) *E* − 0.500 (0.112) *S* − 0.597 (0.234) *A* − 2.044 (0.229) *B* + 1.441 (0.223) *V* N = 27; SD = 0.296; R^2^ = 0.835; F = 21.3 (9)

where N is the number of compounds in the correlation, SD is the standard deviation, R^2^ is the determination coefficient, and F is the Fisher F-statistic. The standard deviations of the correlation constant and of each coefficient are shown in parenthesis. All coefficients are significant at 95% confidence level, except for the excess molar refractivity parameter (*E*), which has a coefficient of close to zero with a high standard deviation. 

The second skin-PAMPA system analyzed is the one based on the membranes developed by Sinkó et al. [13], which are commercially available (Pion Inc, Billerica, MA, USA). In a previous work [39], the log *P*_0_ values of a set of compounds were measured in gradient pH conditions. In the donor compartment, a pH was used in which the compound was in its neutral form, whereas in the acceptor compartment, the buffer was at pH 7.4. The set of compounds, the permeability data, and the descriptors for Equation (1) are shown in Table S1 of the Supplementary Materials. The obtained correlation equation is as follows:

log *P*_e_ (PAMPA-Certramide) = −4.181 (0.088) + 0.064 (0.104) *E* − 0.594 (0.049) *S* − 1.038 (0.080) *A* − 2.269 (0.096) *B* + 1.730 (0.079) *V* N = 45; SD = 0.154; R^2^ = 0.964; F = 210.6 (10)

Although statistics are better for Equation (10), both equations present important similarities: the *e* coefficient is again not statistically significative, and the sign of the rest of coefficients is the same in both equations. Moreover, coefficient values are also similar in magnitude, except for coefficient *a*, which is more negative in the PAMPA-Certramide system. This is, apparently, the main difference between both skin-PAMPA membranes, and it means that the hydrogen bond basicity of the PAMPA-Certramide system, based on cetramides, is lower than the basicity of the silicone-IPM membrane.

### 3.2. Comparison of PAMPA Membranes through the Solvation Parameter Model

In order to identify whether the two skin-PAMPA systems present similarities to other PAMPA membranes, different PAMPA equations have been compiled, essentially via the analysis of Abraham and coworkers [37], in order to compare the coefficients. The evaluated systems, Equation (1) coefficients, and the main constituents of the membranes are presented in Table 1. Systems 1 (PAMPA-Certramide) and 2 (PAMPA-IPM) have equations that have been established in the present work and developed to mimic permeation through the skin. Systems 3–9 were previously evaluated by Abraham [37]. System 3 (PAMPA-BBB) was a membrane initially thought to mimic brain permeation, made of a porcine brain lipid extract. The remainder of systems (4–9) were developed to simulate the HIA; Systems 4 (PAMPA-HDM), 5 (PAMPA-DOPC), and 6 (PAMPA-DS), developed by Avdeef et al. [43] consisted of a filter plate coated with solutions of different natures: n-hexadecane (4), dioleyoylphosphatidylcholine in n-dodecane (5), and a mixture of lecithins in n-dodecane (6); systems 7 (PAMPA P0) and 8 (PAMPA-COS) also developed by Avdeef et al. [4,44], have the same membrane as the PAMPA-DS system (6). However, the PAMPA-P0 (7) LSER equation was carried out using a wider set of data, and the PAMPA-COS (8) system evaluates the permeability of a smaller set of compounds used to develop a method to measure the permeability of highly insoluble compounds. Finally, the PAMPA-P16 system (9), developed by Wohnsland and Faller [38], also consisted of a filter coated with n-hexadecane.

**Figure 1:**
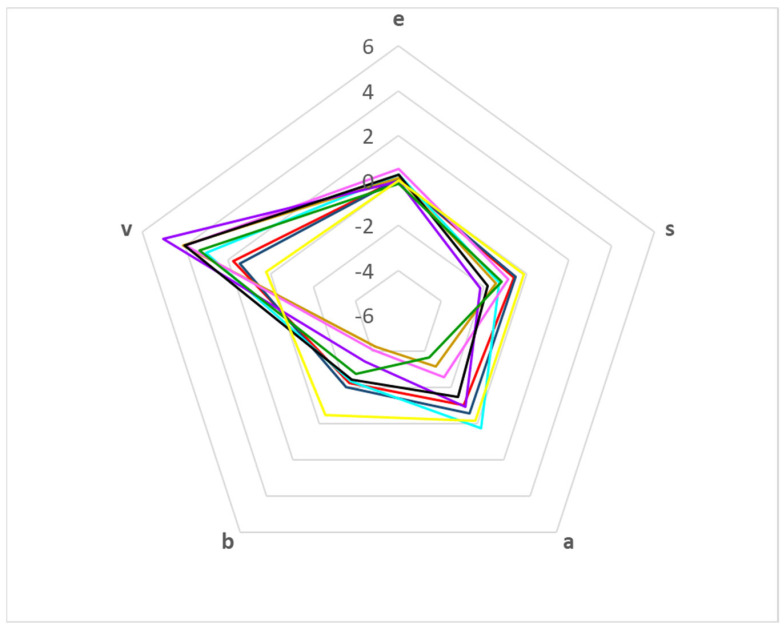
Radial plot according to the coefficient values for the different PAMPA systems: PAMPA-Certramide (1, **—**); PAMPA-IPM (2, **—**); PAMPA-BBB (3, **—**); PAMPA-HDM (4, **—**); PAMPA-DOPC (5, **—**); PAMPA-DS (6, **—**); PAMPA-P0 (7, **—**); PAMPA-COS (8, **—**); PAMPA-P16 (9, **—**).

There are different ways to compare the coefficients of the solvation parameter model. The simplest way is using a radial plot, in which each axis represents a coefficient of Equation (1). Figure 1 shows the radial plot for the different PAMPA systems. Coefficients *e* and *s* are the most similar, being the close to zero *e* coefficient and the slightly negative *s* coefficient. The properties that allow a major discrimination between systems are hydrophobicity (coefficient *v*) and the hydrogen bond properties (coefficients *a* and *b*).

As regards hydrophobicity, all membranes are more hydrophobic than the aqueous phases (indicated by the positive *v* coefficients). Again, the membranes with higher hydrophobicity are those designed for intestinal absorption (with the exception of PAMPA-P16 membrane), whereas the two skin-PAMPA systems seem to be less hydrophobic.

Almost all PAMPA membranes have less hydrogen bond basicity than the aqueous phases, as the coefficient *a* is negative, except for the PAMPA-BBB system (3). However, a direct comparison between the systems show that the skin-PAMPA membranes (1 and 2) have higher hydrogen bond basicity than most of the membranes designed for intestinal absorption. Only the PAMPA-P16 system (9) shows a slightly higher basicity than the skin membranes.

Skin-PAMPA systems also present differences regarding their hydrogen bond acidity (*b* coefficient). In absolute values, the *b* coefficient is the most important coefficient in the skin systems, even higher than the *v* value. However, for the blood–brain (3) and intestinal absorption (4–9) PAMPA systems, the *v* coefficient tends to be similar or higher than *b*. System 9 presents an unexpectedly low absolute value of *b* and *v* coefficients compared to system 4, which has the same type of membrane. However, the coefficient values for system 9 must be taken with caution, as the statistical correlation parameters are not very good (R^2^ = 0.558, S = 0.325) [26].

Another way to visualize the similarities between the whole set of systems is through the distance parameter (*D*′) [28]. *D*′ evaluation can be carried out using pairs of systems, although a dendrogram offers a general overview on how close the systems are, according to their distance and how can they be grouped.

**Figure 2:**
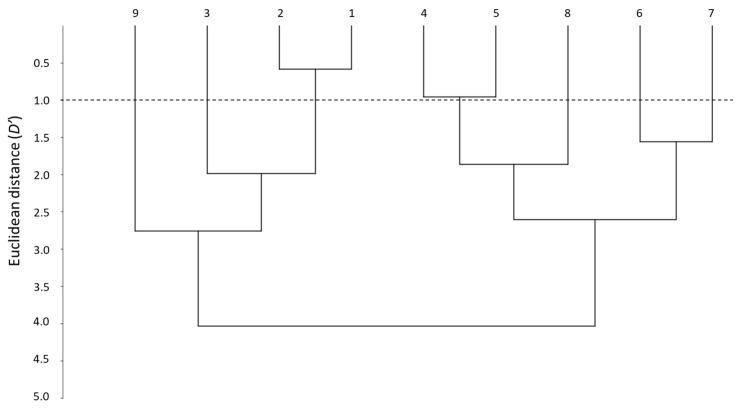
Dendrogram between the different PAMPA systems.

Figure 2 shows the dendrogram obtained when the nine PAMPA systems are compared. The smallest *D*′ is between systems 1 and 2, which forms a first clear cluster (*D*′ < 1). This means that the two skin-PAMPA membranes, despite having different natures, behave in a very similar way. Another cluster can be observed between systems 4 and 5, also with a relatively low distance (*D*′ ~ 1). Both systems have membranes coated with solvents of the same nature—system 4 with n-hexadecane and system 5 n-dodecane—although the latter contains also a relatively small amount of DOPC (2%). Systems 6 and 7 have the same membrane and only the number of solutes analyzed is different, so they also appear to be grouped in the dendrogram, although with a larger distance (*D*′ ~ 1.5). Despite having the same membrane as systems 6 and 7, system 8 appears at a higher distance (*D*′ > 2.5), being in fact more similar to systems 4 and 5 (joining them at *D*′ close to 2). Again, system 9 and system 4, both with a hexadecane-coated membrane, appear very far one from each other. However, as indicated earlier, the data for the correlations of system 9 are very poor, and thus, the values of the coefficients and distances do not seem very trustworthy. System 3 was developed for blood–brain distribution, and it is expected to be clearly different from the other PAMPA systems. This is true for the HIA-PAMPA systems (4 to 9), but surprisingly, system 3 is relatively close to skin-PAMPA (*D*′ ~ 2).

**Figure 3:**
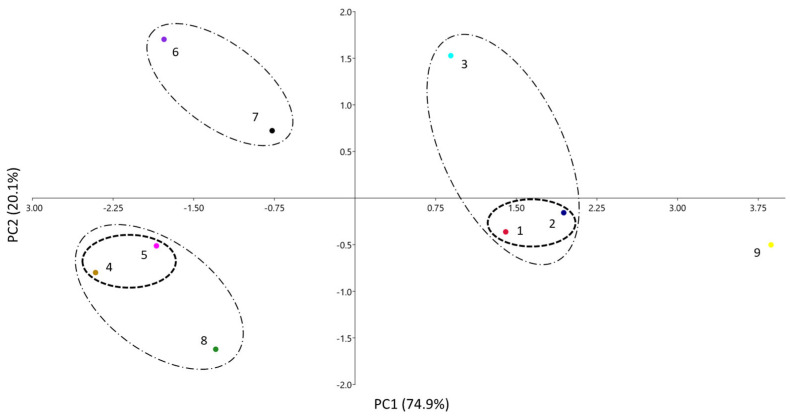
PCA analysis of the different PAMPA systems. Clusters with *D*′ < 1.0 (**– – –**) and clusters with *D*′ between 1 and 2 (– · –).

A third visual way to compare the similarities between the systems is through a principal component analysis (PCA). In this analysis, the number of variables (initially the five coefficients of Equation (1) is reduced, so that the variance of the system can be explained via new variables (principal components, PC), which are orthogonal to each other and have different contributions of the original variables. Figure 3 shows the results of the PCA for the PAMPA systems of Table 1, where they are plotted according to the two main PCs. As only two PCs can explain 95% of the data total variance, results are expected to be quite similar to the ones obtained via the dendrogram and the radial plot.

According to Table 2, the main contributions to PC1 are coefficients *a*, *b*, and *s* (the latter at a minor level) positively, and *v* negatively, respectively. For PC2, the main contribution is *a* (positively) and, to a minor extent, *v* (positively) and *s* (negatively). Notice that coefficient *e*, very similar and close to zero in all instances (see Table 1 or Figure 1), does not play any role in the differentiation of the systems, and coefficient *s* makes only a minor contribution. This was already seen in the radial plot analysis. A certain parallelism can be identified also between Figure 2 and Figure 3: the left part of the dendrogram has the systems located in the area of positive PC1, whereas in the right part, the systems with negative PC1 can be found, all of them grouped in a similar way in both analyses. Therefore, in the PCA, the systems have been grouped according to distances at two *D*′ levels: clusters with *D*′ < 1.0 and clusters with *D*′ between 1 and 2.

The first cluster is composed of the two systems designed to emulate skin (1 and 2), which are very close one to each other (*D*′ < 1). They are located in the area of positive PC1, as they have the fewer negative *a* and *b* coefficients and a relatively low *v* one, compared to the other systems (except for systems 3 and 9). Systems 4 and 5 are also very close and they form another clear cluster with *D*′ < 1 with negative PC1 and PC2 values. 

In the second level of clustering (1 < *D*′ < 2), it can be observed that the cluster between systems 6 and 7 has low PC1 and high PC2 values. Additionally, the supracluster in system 8 with the clusters 4 and 5 can be observed in the same area, but with a lower value of PC2 because of its lower *a* coefficient (the most negative of all systems). The last supracluster is that of system 3 (BB-PAMPA) with the skin-PAMPA cluster (1 + 2 systems) and the three systems with positive PC1. The main difference is the highest PC2 value of system 3, caused by its high *a* coefficient, the only positive *a* coefficient of all the PAMPA membranes studied. System 9 is located far from the other systems and does not display significant similarities to them. As explained, this system has very different coefficients from the others and displays poor correlation.

Two conclusions that can be drawn from the clustering analysis: firstly, that hydrophobicity (*v* coefficient) and hydrogen bond interactions (*a* and *b* coefficients) are the properties that allow the differentiation between the PAMPA systems; and secondly, that the systems are somehow different, which is of special interest for the emulation of relevant biological processes.

### 3.3. Evaluation of the Ability of Different Pampa Systems to Emulate Biological Processes

In the former study of Abraham and coworkers [37], they concluded that none of the PAMPA systems were adequate for emulating skin permeability, skin partitions, or brain permeability. Of the systems thought to model HIA, only system 9 seemed to be good enough for this purpose. However, they did not included skin-PAMPA systems in their analysis because these were not available at that time.

This analysis was repeated, but including the two new skin-PAMPA systems (1 and 2), and the similarities and differences between the PAMPA and the relevant biological processes were again compared. The selected biological systems are the skin permeability (10) [27], the water–skin partition (11) [28], the HIA (12) [26], the blood/serum/plasma-brain partition (13) [30], and saline–brain permeation (14) [29]. The coefficients for the biological processes are also shown in Table 1. *D*′ distances between PAMPA systems and biological systems are presented in Table 3. System 9 was excluded from the study because it is very different from the others (see Figure 1, Figure 2 and Figure 3) and has poor statistics and very low correlation coefficients, which make it untrustworthy and poorly selective.

Results in Table 3 confirm that none of the PAMPA systems studied by Abraham are good enough to evaluate any of the biological processes because all *D*′ distances are much larger than 1. However, the two new skin-PAMPA systems (1 and 2) have *D*′ distances lower than 1 in comparison to some of the studied biological processes. It is clear that they are able to emulate skin permeation (system 10) well, presenting *D*′ distances of 0.6–0.7. They are also able to emulate water–skin partitions (system 11), especially the silicone oil and IPM membrane (system 2), which shows a distance of about 0.8 to the water–skin partition. This is not surprising because the solute–solvent interactions for skin partition and permeation are very similar. In fact, both processes are very close at *D*′ = 0.60. More surprising is that skin-PAMPA membranes are able to emulate brain perfusion quite well (system 14), at *D*′ = 0.7–0.9, which is better than the PAMPA membrane designed for blood–brain measurements (system 3). As already explained in the dendrogram and PCA clustering, this membrane is not far from the skin-PAMPA membranes but not close enough to brain measurements (*D*′ values of 1.9 and 3.1 for the brain perfusion and the blood–brain partition, respectively). Although the skin-PAMPA membranes are closer to the blood–brain partition than the original blood–brain membranes, they are not close enough to emulate it (*D*′ ≈ 1.6–2.0). New PAMPA membranes should be designed and studied to emulate the blood–brain partition and human intestinal absorption.

## 4. Conclusions

The comparison of the coefficients of partition systems of different natures characterized through the solvation parameter model is a good method to see how important the similarities and dissimilarities between them are.

The new equations obtained for two PAMPA systems intended for the simulation of permeation through the skin has allowed a comparison of these membranes to other existing PAMPA membranes. This evaluation has revealed that the hydrogen bond parameters and the hydrophobicity, although especially the hydrogen bond basicity, are the properties that allow the best differentiation between the membranes. The different analyses indicate that the two skin-PAMPA membranes are very similar in terms of permeation despite their different natures. Differences can also be observed among the membranes created to emulate the HIA.

A further comparison between permeability in the PAMPA membranes and certain biological processes point out that the two skin-PAMPA membranes are effective for emulating skin permeation. They are also able to emulate the water–skin partition well, which is a process quite similar to skin permeation. Surprisingly, they can also emulate brain perfusion quite well and the blood–brain partition much better than the PAMPA membranes designed for this purpose.

## Figures and Tables

**Table 1:** Compilation of PAMPA and biological systems characterized by Equation (1).

System		*e*	*s*	*a*	*b*	*v*	PAMPA Membrane Components	Ref
1	PAMPA-Certramide	0.064	−0.594	−1.038	−2.269	1.73	certramide, cholesterol, stearic acid, and silicone oil	[13]
2	PAMPA-IPM	0.081	−0.5	−0.597	−2.044	1.441	70% silicone oil and 30% IPM	[12]
3	PAMPA-BBB	0.25	−1.29	0.25	−2.37	3.03	10% (*w*/*v*) porcine brain lipid extract in alkane	[10]
4	PAMPA-HDM	0.106	−1.44	−3.18	−4.24	4.09	n-hexadecane	[43]
5	PAMPA-DOPC	0.51	−0.86	−2.57	−4.07	3.99	2% *w*/*v* dioleyoylphosphatidylcholine in n-dodecane	[43]
6	PAMPA-DS	−0.026	−2.17	−0.951	−3.45	5.01	20% (*w*/*v*) of a lecithin mixture in n-dodecane	[43]
7	PAMPA-P0	0.25	−1.84	−1.48	−2.46	4.02	20% (*w*/*v*) of a lecithin mixture in n-dodecane	[4]
8	PAMPA-COS	−0.13	−1.17	−3.65	−2.76	3.33	20% (*w*/*v*) of a lecithin mixture in n-dodecane.	[44]
9	PAMPA-P16	0	−0.121	−0.188	−0.479	0.194	n-hexadecane	[38]
10	Skin permeation	0.137	−0.604	−0.338	−2.428	1.797		[27]
11	Skin partition	0.341	−0.206	−0.024	−2.178	1.85		[28]
12	HIA	0	0	−0.284	−0.343	0.262		[26]
13	Blood–brain partition	0.221	−0.604	−0.641	−0.681	0.635		[30]
14	Saline–brain permeation	−0.047	−0.876	−0.719	−1.571	1.767		[29]

**Table 2:** Contribution of each coefficient to the main principal components.

Coefficient	Analysis of PAMPA Systems (Figure 3)	
	**PC1**	**PC2**
*e*	−0.020	0.036
*s*	0.233	−0.325
*a*	0.466	0.828
*b*	0.504	0.006
*v*	−0.688	0.454

**Table 3:** *D*′ values between the PAMPA systems (1–8) and the biological ones (10–14). In bold, *D*′ values equal or lower than 1.

	1	2	3	4	5	6	7	8
**10**	**0.72**	**0.60**	1.53	4.16	3.56	3.77	2.79	3.72
**11**	1.13	**0.82**	1.64	4.56	3.89	4.06	3.10	4.10
**12**	2.61	2.15	3.71	6.35	5.83	6.11	4.85	5.29
**13**	1.98	1.59	3.14	5.64	5.16	5.42	4.11	4.59
**14**	**0.83**	**0.71**	1.85	4.35	3.86	3.97	2.73	3.54

## Data Availability

Data available in the Supplementary Materials.

## Supplementary Materials

The following supporting information can be downloaded at: https://www.mdpi.com/article/10.3390/membranes13070640/s1, Table S1: Permeability and descriptor values for the compounds tested in the certramide PAMPA system (PAMPA-Certramide); Table S2: Permeability and descriptor values for the compounds tested in the 70% silicon–30% IPM PAMPA system (PAMPA-IPM).
