# Supplementary material for: Evaluation of the Ability of PAMPA Membranes to Emulate Biological Processes through the Abraham Solvation Parameter Model
Source: Membranes (Basel). 2023 Jun 30;13(7):640. doi: 10.3390/membranes13070640 (PMC10385989; doi:10.3390/membranes13070640)
Supplement: Supplementary file 1 [file membranes-13-00640-s001.zip › membranes-2478824-supplementary.pdf]

Prediction of skin permeation through parallel artificial membrane permeability assays (PAMPA). Evaluation through the Abraham solvation parameter model.

Sara Soriano-Meseguer[1], Elisabet Fuguet[1,2,3,*], Adriana Port[4], Martí Rosés[1,2]

[1] Departament d'Enginyeria Química i Química Analítica, Universitat de Barcelona (UB), Martí i Franquès 1-11, 08028 Barcelona, Spain. sara.sorioanomeseguer@gmail.com; elifuguetj@ub.edu; marti.roses@ub.edu

[2] Institut de Biomedicina de la Universitat de Barcelona (IBUB), Universitat de Barcelona (UB), av. Diagonal, 643, 08028 Barcelona, Spain.

[3] Serra Húnter Programme, Generalitat de Catalunya, 08002 Barcelona, Spain.

[4] Welab Barcelona, Parc Científic de Barcelona, Baldiri Reixac, 4-8, 08028 Barcelona, Spain. aport@welab.barcelona

Supplementary Information

Table S1. Permeability and descriptor values for the compounds tested in the certramide PAMPA system (PAMPA-Certramide).

	log P_0	*E*	*S*	*A*	*B*	*V*
2,4-dichlorophenol	-3.92	0.960	0.82	0.54	0.17	1.0199
2-hydroxybenzoic acid	-4.42	0.900	0.85	0.73	0.37	0.9904
2-isopropyl-5-methylphenol (thymol)	-4.01	0.822	0.80	0.43	0.44	1.3387
2-nitro-*p*-phenylenediamine	-5.24	1.525	2.05	0.35	0.70	1.0902
3-methylphenol (*m*-cresol)	-4.33	0.822	0.88	0.57	0.34	0.9160
4-amino-2-nitrophenol	-4.59	1.360	1.50	0.30	0.66	1.0491
4-chlorophenol	-4.27	0.915	1.08	0.67	0.20	0.8975
4-ethylphenol	-4.19	0.800	0.90	0.55	0.36	1.0569
4-hiyroxybenzyl alcohol	-5.85	0.998	1.30	0.86	0.79	0.9747
4-hydroxymethyl phenylacetate	-5.07	0.908	1.46	0.59	0.68	1.2722
4-hydroxyphenyl acetamide	-6.07	1.180	2.08	0.84	0.94	1.1724
4-nitrophenol	-4.91	1.070	1.72	0.82	0.26	0.9493
5,5-diethylbarbituric acid (barbital)	-5.75	1.030	1.00	0.58	1.12	1.3739
5-ethyl-5-phenylbarbituric acid (phenobarbital)	-6.05	1.630	1.72	0.71	1.18	1.6999
5-fluorouracil	-5.77	0.720	0.84	0.57	1.02	0.7693
8-methoxypsoralen	-4.30	1.611	1.70	0.00	0.80	1.4504
aminopyrine	-5.66	1.680	1.74	0.00	1.60	1.8662
aniline	-4.55	0.955	0.96	0.26	0.41	0.8162
antipyrine	-5.63	1.300	1.83	0.00	1.37	1.4846
atrazine	-4.67	1.220	1.29	0.17	1.01	1.6196
benzoic acid	-4.81	0.730	0.90	0.59	0.40	0.9317
benzyl nicotinate	-4.15	1.262	1.38	0.00	0.85	1.6393
caffeine	-5.45	1.500	1.82	0.08	1.25	1.3632
capsaicin	-4.63	1.250	2.19	0.57	1.45	2.5971
catechol	-5.39	0.970	1.10	0.88	0.47	0.8338
corteloxone	-5.45	1.910	3.45	0.36	1.60	2.7389
corticosterone	-5.59	1.860	3.43	0.40	1.63	2.7389
cortisone	-6.09	1.960	3.50	0.36	1.87	2.7546
dexamethasone	-6.25	2.040	3.51	0.71	1.92	2.9132
diclofenac	-3.76	1.810	1.85	0.55	0.77	2.0250
digitoxin	-6.38	3.460	5.63	1.33	4.35	5.6938
estradiol	-4.15	1.800	1.77	0.86	1.10	2.1988
estriol	-6.07	1.970	1.74	1.06	1.63	2.2575
fluocinonide	-5.38	1.950	2.48	0.31	2.51	3.4601
flurbiprofen	-3.65	1.440	1.45	0.62	0.76	1.8389
griseofulvin	-5.28	1.750	2.64	0.00	1.44	2.3947
hydrocortisone	-6.17	2.030	3.49	0.71	1.90	2.7976
hydroquinone	-5.87	1.063	1.27	1.06	0.57	0.8338
hydroxiprogesterone	-4.70	1.640	3.35	0.25	1.31	2.6802
ibuprofen	-3.59	0.730	0.70	0.57	0.79	1.7771
indomethacine	-4.35	2.240	1.47	0.58	1.43	2.5299
isoquinoline	-4.19	1.211	1.00	0.00	0.54	1.0443
ketoprofen	-4.64	1.650	2.26	0.55	0.89	1.9779
ketorolac	-5.05	1.600	2.03	0.65	1.05	1.8712

Methyl 4-hydroxybenzoate	-4.88	0.930	1.46	0.71	0.46	1.1313
methyl phenyl ether	-4.34	0.708	0.75	0.00	0.29	0.9160
naproxen	-4.18	1.510	2.02	0.60	0.67	1.7821
o-phenylenediamine	-5.41	1.260	1.40	0.24	0.73	0.9160
o-toluidine	-4.13	0.966	0.92	0.23	0.45	0.9571
prednisolone	-6.42	2.210	3.10	0.71	1.92	2.7546
progesterone	-4.13	1.450	3.29	0.00	1.14	2.6215
pyridine	-4.49	0.631	0.84	0.00	0.52	0.6753
pyroxicam	-4.69	2.560	2.90	0.17	1.49	2.2500
testosterone	-4.52	1.540	2.56	0.32	1.17	2.3827
warfarin	-4.63	1.980	1.88	0.29	1.57	2.3077

Table S2. Permeability and descriptor values for the compounds tested in the 70% silicon – 30% IPM PAMPA system (PAMPA-IPM).

	log P_0	*E*	*S*	*A*	*B*	*V*
2-amino-4-nitrophenol	-4.84	1.415	1.95	1.01	0.43	1.0491
2-naphthol	-4.19	1.52	1.08	0.61	0.4	1.1441
2-nitro-*p*-phenylendiamine	-4.94	1.525	2.05	0.35	0.7	1.0902
4-bromophenol	-3.9	1.08	1.17	0.67	0.2	0.9501
4-chlorophenol	-4.1	0.915	1.08	0.67	0.2	0.8975
4-ethylphenol	-4.05	0.8	0.9	0.55	0.36	1.0569
4-nitrophenol	-4.33	1.07	1.72	0.82	0.26	0.9493
antipyrine	-6.2	1.32	1.5	0	1.48	1.4846
atrazine	-4.21	1.51	1.17	0.32	0.96	1.6196
benzyl nicotinate	-4.26	1.262	1.6	0	0.8	1.6393
caffeine	-5.63	1.5	1.72	0.05	1.28	1.3632
corticosterone	-5.06	1.86	3.43	0.4	1.63	2.7389
dexamethasone	-5.75	2.04	3.51	0.71	1.92	2.9131
diclofenac	-4.33	2.275	1.22	0.63	0.96	2.025
ephedrine	-4.82	0.916	0.76	0.21	1.21	1.4385
hydrocortisone	-5.94	2.03	3.49	0.71	1.9	2.7976
indomethacin	-4.2	2.236	1.35	0.57	1.57	2.5299
isoquinoline	-4.05	1.211	1	0	0.47	1.0443
ketoprofen	-4.25	1.65	2.26	0.55	0.89	1.9779
lidocaine	-4.35	1.01	1.5	0.12	1.21	2.0589
methyl nicotinate	-4.32	0.71	1.13	0	0.71	1.0315
naproxen	-4.11	1.51	1.98	0.6	0.68	1.7821
nicotine	-4.58	0.865	0.92	0	1.08	1.371
phenobarbital	-5.15	1.63	1.8	0.73	1.15	1.6999
phenol	-4.14	0.805	0.89	0.6	0.3	0.7751
piroxicam	-4.35	2.84	1.91	0.55	1.82	2.25
progesterone	-4.56	1.45	3.29	0	1.14	2.6215
resorcinol	-5.62	0.98	1.11	1.09	0.52	0.8338
salicylic acid	-4.16	0.89	0.84	0.7	0.4	0.9904
testosterone	-4.11	1.54	2.59	0.32	1.19	2.3827
thymol	-4.34	0.822	0.79	0.52	0.44	1.3387
